# Construction of a High-Density *Paulownia* Genetic Map and QTL Mapping of Important Phenotypic Traits Based on Genome Assembly and Whole-Genome Resequencing

**DOI:** 10.3390/ijms242115647

**Published:** 2023-10-27

**Authors:** Yanzhi Feng, Chaowei Yang, Jiajia Zhang, Jie Qiao, Baoping Wang, Yang Zhao

**Affiliations:** 1 Research Institute of Non-Timber Forestry, Chinese Academy of Forestry, Zhengzhou 450003, China; fyz617912@126.com (Y.F.); yangcw010@126.com (C.Y.); jiajiazhang@caf.ac.cn (J.Z.); qiaoj3715@163.com (J.Q.); 2 Key Laboratory of Non-Timber Forest Germplasm Enhancement & Utilization of National Forestry and Grassland Administration, Zhengzhou 450003, China

**Keywords:** *Paulownia*, genome, whole-genome resequencing, genetic map, important phenotypic traits, quantitative trait locus

## Abstract

Quantitative trait locus (QTL) mapping based on a genetic map is a very effective method of marker-assisted selection in breeding, and whole-genome resequencing is one of the useful methods to obtain high-density genetic maps. In this study, the hybrid assembly of Illumina, PacBio, and chromatin interaction mapping data was used to construct high-quality chromosomal genome sequences of *Paulownia fortunei*, with a size of 476.82 Mb, a heterozygosity of 0.52%, and a contig and scaffold N50s of 7.81 Mb and 21.81 Mb, respectively. Twenty scaffolds with a total length of 437.72 Mb were assembled into 20 pseudochromosomes. Repeat sequences with a total length of 243.96 Mb accounted for 51.16% of the entire genome. In all, 26,903 protein-coding gene loci were identified, and 26,008 (96.67%) genes had conserved functional motifs. Further comparative genomics analysis preliminarily showed that the split of *P*. *fortunei* with *Tectona grandis* likely occurred 38.8 (33.3–45.1) million years ago. Whole-genome resequencing was used to construct a merged genetic map of 20 linkage groups, with 2993 bin markers (3,312,780 SNPs), a total length of 1675.14 cm, and an average marker interval of 0.56 cm. In total, 73 QTLs for important phenotypic traits were identified (19 major QTLs with phenotypic variation explained ≥ 10%), including 10 for the diameter at breast height, 7 for the main trunk height, and 56 for branch-related traits. These results not only enrich *P*. *fortunei* genomic data but also form a solid foundation for fine QTL mapping and key marker/gene mining of *Paulownia*, which is of great significance for the directed genetic improvement of these species.

## 1. Introduction

So far, a large number of high-quality plant genomes have been published, such as *Vernicia fordii* [1], *Diospyros oleifera* Cheng [2], Tibetan hulless barley [3], etc. Reference genomes of various individuals can provide new insights into genomic structure and evolution, genetic diversity, and phylogeny [4]. For example, the successful assembly and accurate comparison of three *Capsicum* genomes made possible a lineage-specific evolution study of gene families [5]; the phylogenetic analysis of *Salix matsudana* Koidz based on its genome sequence and comparative genomics analysis would be helpful to discover its evolutionary history [6]; and the completion of the reference genome of *V. fordii* laid a theoretical foundation for understanding its genomic evolution and speculating the reason for the high oil content of tung seeds [1]. The first draft of the *Paulownia fortunei* genome with high heterozygosity (2.03%) was released in 2021 [7]. However, as this species has a wide distribution range, abundant intraspecific resources, and many variation types, a superior individual of *P. fortunei* was used for genome sequencing and assembly to reduce the heterozygosity and improve *P. fortunei* genomic data.

Quantitative trait locus (QTL) analysis based on genetic linkage maps can not only identify closely linked molecular markers but also determine the locations of genes involved in the regulation of complex quantitative traits [8,9]. Following the successful construction of the first map of *Petunia* in 1994 [10], many genetic maps have also been constructed for other plant species [10,11,12]. Following amplified fragment length polymorphisms (AFLPs) and simple sequence repeats (SSRs), single nucleotide polymorphisms (SNPs) are considered ideal markers for constructing high-density genetic maps [10]. In a previous study, 204,361 SNPs were developed from the F1 generation *Ginkgo biloba*, among which 12,263 were used to construct a high-density genetic map. The map was 9.93 cm shorter than those constructed based on AFLPs, SSRs, and sequence-related amplified polymorphisms, with 10,000 more SNPs employed [10]. A large number of high-density genetic maps have been successfully constructed for *Prunus mume* [13], *Osmanthus fragrans* [14], and *Paeonia suffruticosa* [15]. SNPs developed from whole genomes can lay a solid foundation for finely mapping important plant traits and populational evolution [16].

Different plants have unique architectures and corresponding functions [17]. The ideal plant architecture achieves high and stable yields by increasing the density of planting per unit area [17,18]. Plant architecture is principally affected by the spatial patterning of branches, e.g., internode length, branch angle, and branch number [19,20]. Branching is a complex biological phenomenon resulting from the interaction between genes, hormones, and the environment [21]. Branching parameters, particularly branch angle, determine the architecture [22], which is also a fundamental factor leading to the variety of plant architectures. Planting density is a principal environmental factor affecting the architecture [17]. Therefore, the branch angle is also an important trait of great interest to breeders. However, although there has been much research on the branch angles of crops and fruit trees [17,20,23,24], less is known about other trees [25]. To date, there have been no reports on *Paulownia* branch characteristics. Therefore, we carried out QTL mapping based on a genetic linkage map with the growth and branching of important phenotypic traits jointly controlled by multiple mini-effect genes.

*Paulownia* is one of the fastest-growing timber trees in the world and is widely distributed in subtropical and warm temperate areas [26,27]. *Paulownia* is planted mainly for producing timber, improving the microclimate of intercropping systems, and reducing soil erosion [27,28,29]. *Paulownia* is native to China and has been introduced to America, Italy, Brazil, Argentina, and other countries, where its wood is usually used in the production of furniture, plywood, and paper [27]. There are 11 species of the genus *Paulownia*, including 4 original species and 7 derived species. *P. fortunei* is one of four original species with a fast growth rate, relatively large branch angle, and strong natural trunk extension ability, while *Paulownia catalpifolia,* with a relatively slow growth rate and small branch angle, is one of seven derivative *Paulownia* species.

In the present study, the *P. fortunei* genome obtained using a combination of short-read (Illumina) and long-read (PacBio) sequencing and assembly technologies was used as the reference genome. Whole-genome resequencing data of F1 hybrid populations of *P. fortunei* and *P. catalpifolia* were used to construct a genetic map. The loci that significantly correlated with the important phenotypic traits of *Paulownia* were located by combining the phenotypic data, which will lay a solid foundation for excavating potential key candidate markers/genes. Our findings will greatly facilitate an analysis of the genetic basis of *Paulownia* growth and branching characteristics and provide new insights for the directed genetic improvement of *Paulownia* architecture.

## 2. Results

### 2.1. Sequencing, Assembly, and Annotation of the P. fortunei Genome

**Figure 1:**
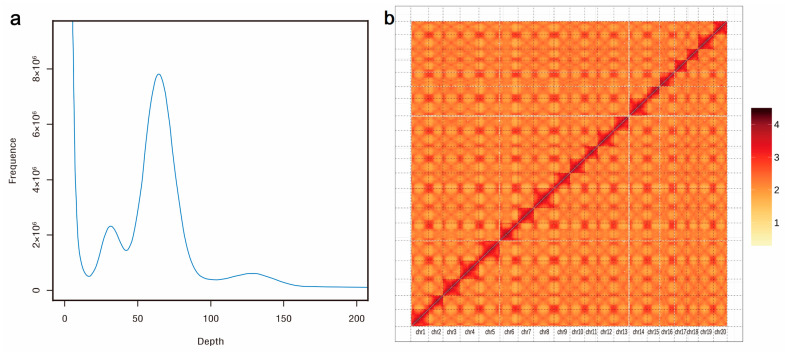
The 17-mer frequency distribution and Hi-C heatmap of *P. fortunei* genomes: (**a**) 17-mer frequency distribution of the genome, the *X*-axis is the *K*-mer depth, and the *Y*-axis represents the frequency of the *K*-mer for a given depth; (**b**) The Hi-C heatmap of the genome. A total of 20 chromosomes were numbered from asm_0 to asm_19.

**Figure 2:**
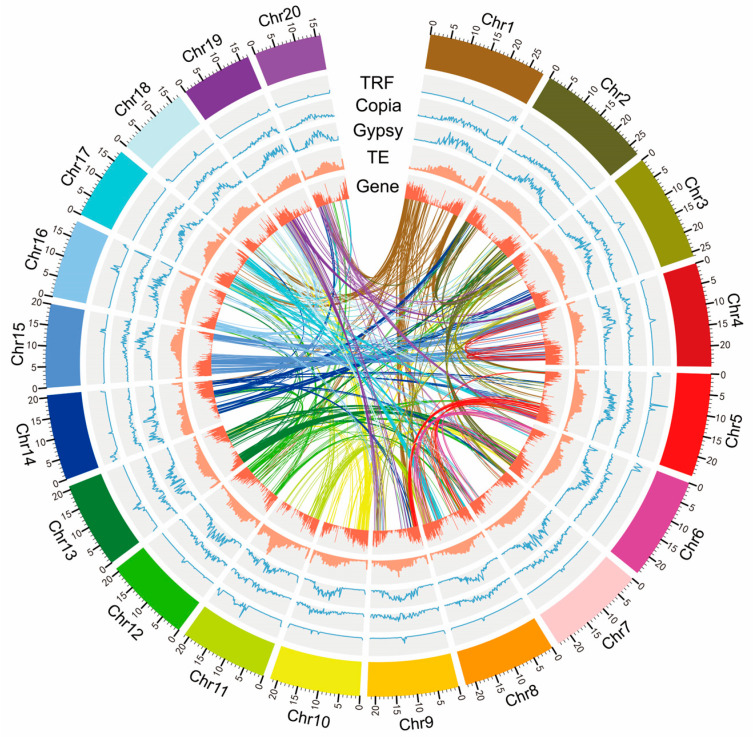
*P. fortunei* genomes: Circular diagram showing genetic collinearity among *P. fortunei* chromosomes. The concentric circles (from inside to outside) are as follows: gene density, transposable element (TE) density, Gypsy density, Copia density, tandem repeats finder (TRF) density, and genome collinear blocks, which are connected by curved lines of the same color for each element. All distributions are drawn in a 200 kb window size.

A total of 47.73 Gb (90.33× coverage) of Illumina short cleaned reads were generated for *K-mer* analysis and base correction; 233 Gb (440.95× coverage) of long sequencing reads were finally obtained from the PacBio platform, and 116 Gb of high-throughput chromosome conformation capture (Hi-C) sequence data (219.53× coverage) were ultimately obtained. The genome size of *P*. *fortunei* was estimated to be 528.41 Mb with 0.52% heterozygosity and a repeat sequence ratio of 50.98% (Figure 1a). By combining short cleaned reads, long sequencing reads, and Hi-C sequence data, a draft genome of *P. fortunei* with a total length of 476.82 Mb, a contig N50 length of 7.81 Mb, and a scaffold N50 length of 21.81 Mb was constructed. As a diploid species (2n = 40) [7], 20 pseudochromosomes with a total length of 437.72 Mb (accounting for 91.80% of the entire genome) were finally obtained (Table 1 and Figure 1b). A map connecting homologous regions of the *P*. *fortunei* genome is shown in Figure 2. The benchmarking universal single-copy orthologs (BUSCO) assessment indicated that 93.50% of the complete BUSCO genes (1440) were identified. By contrast, 240 (96.77%) of 248 core evolutionarily conserved genes were detected and assembled in the genome using the core eukaryotic genes mapping approach (CEGMA), confirming the high completeness of the genome assembly. The BWA evaluation indicated that 95.25% of the reads were aligned to the genome, and the coverage rate was about 97.56%.

**Figure 3:**
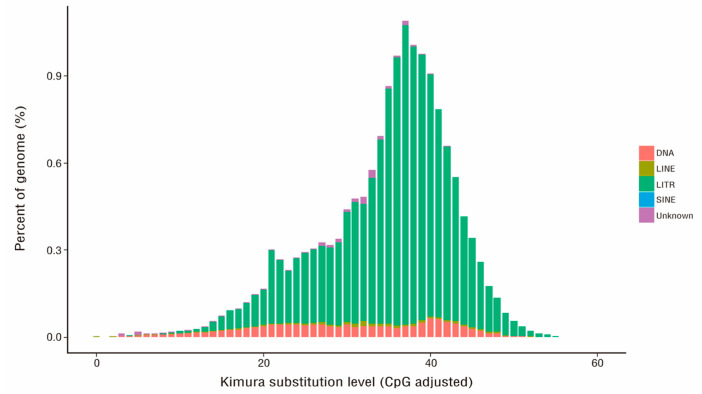
Distribution of the divergence rate of each type of repetitive element in the *P. fortunei* genome. The divergence rate was calculated between the identified TE elements in the genome using the homology-based method and the consensus sequence in the Repbase.

A total of 243.96 Mb of non-redundant repeat sequences annotated using tandem repeats finder (TRF) (72.78 Mb, 15.26% of the genome), RepeatMasker (236.00 Mb, 49.49% of the genome), and RepeatProteinMask (43.02 Mb, 9.02% of the genome) were finally obtained, accounting for 51.16% of the entire *P. fortunei* genome. The long terminal repeats formed the most abundant category of transposable elements (TEs), accounting for 34.41% of the entire genome and 69.02% of the combined TEs. The Copia- and Gypsy-like long terminal repeats covered 51.48 Mb and 73.53 Mb, accounting for 10.80% and 15.42% of the assembled genome, respectively. Next were DNA transposons and long interspersed nuclear elements, which accounted for 1.42% and 0.36% of the genome, respectively (Figure 3). GlimmerHMM predicted the largest number of genes (46,150), and the average transcript and coding sequence lengths were 7930.75 bp and 748.01 bp, respectively. SNAP (40,819) and Geneid (37,118) yielded average transcript lengths of 3511.44 bp and 5004.03 bp, respectively, followed by Augustus (28,326) and GENSCAN (25,949). In all, 26,903 protein-coding genes with an average transcript size of 3921.04 bp were predicted for the entire genome. The databases with the most annotated genes were NR (25,951; 96.46%) and InterProScan (25,273; 93.94%), followed by SwissProt (21,905; 81.42%), Pfam (21,309; 79.21%), the Kyoto Encyclopedia of Genes and Genomes (KEGG) (20,911; 77.73%), and Gene Ontology (GO) (15,648; 58.16%). Overall, 96.67% (26,008) of the protein-coding genes were successfully annotated for conserved functional motifs or functional terms (Table 2). Overall, 267 miRNAs, 513 tRNAs, 1306 rRNAs, and 1194 snRNAs were discovered in the *P. fortunei* genome, and their average lengths were 124.29 bp, 74.93 bp, 149.61 bp, and 111.21 bp, respectively.

### 2.2. Comparative Genome Analysis

**Figure 4:**
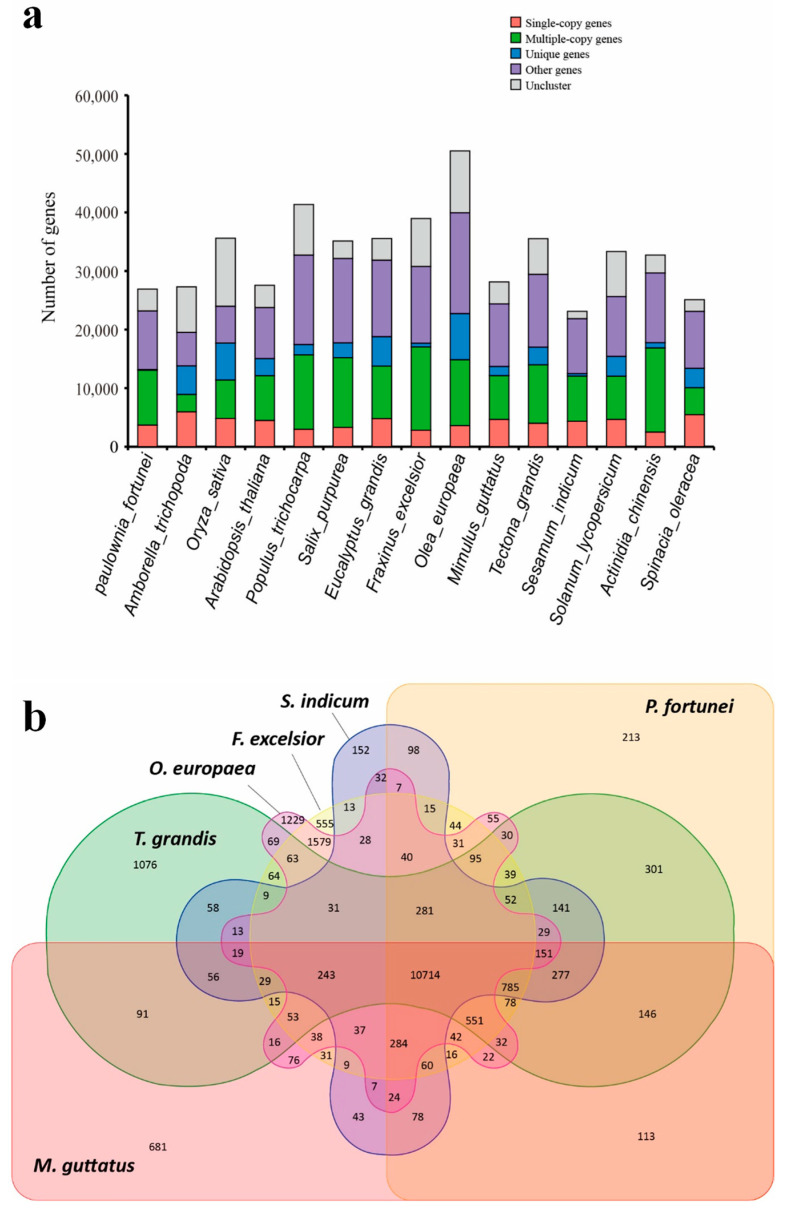
Gene family clustering of *P. fortunei* with other plants: (**a**) the distribution of genes (single-copy genes, multiple-copy genes, unique, other, and uncluster genes number) in 15 different species; (**b**) common and unique gene families between *Paulownia fortunei*, *Mimulus guttatus*, *Tectona grandis*, *Sesamum indicum*, *Olea europaea*, and *Fraxinus excelsior*.

**Figure 5:**
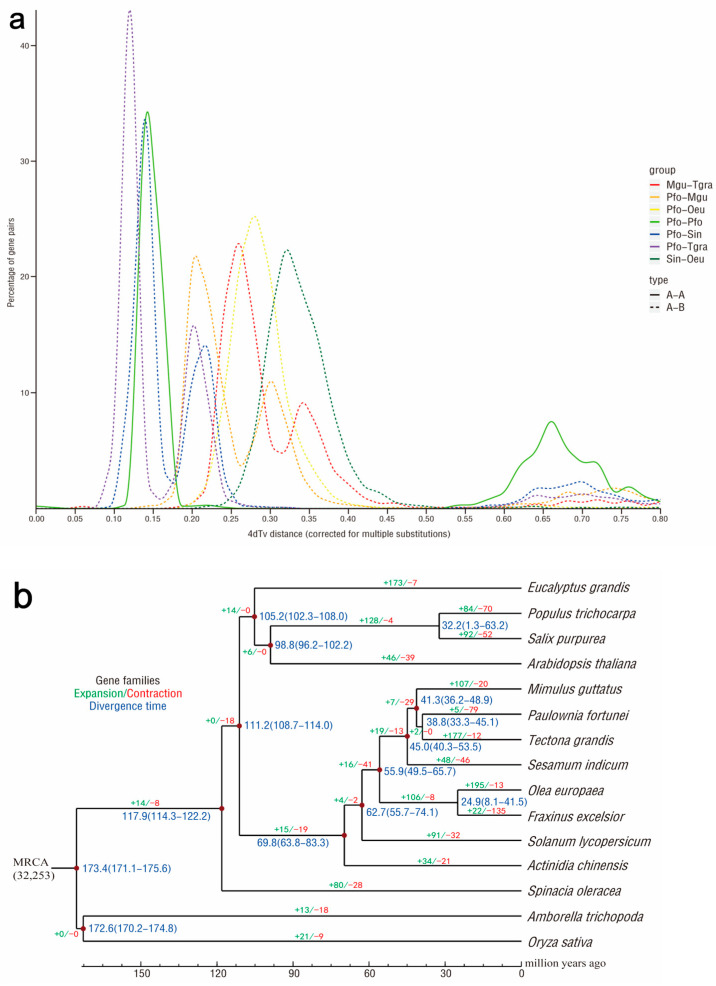
Comparative analyses of *P. fortunei* with other plants: (**a**) whole genome duplication (WGD) events detected in the genomes of *P. fortunei*; (**b**) gene family expansions, contractions, and estimation of divergence time in *P. fortunei* and 14 other species.

A total of 32,270 gene families were found in the 15 species through comparative genomic analysis, among which there were 7040 common gene families and 514 common single-copy gene families, respectively (Figure 4a). Comparison with *Mimulus guttatus* and four other species indicated that 286 genes (213 gene families) were specific to the *P. fortunei* genome (Figure 4b). These unique genes of *P. fortunei* were annotated to biological processes such as the abscisic acid-activated signaling pathway, response to hormone, and defense responses, as well as molecular functions such as abscisic acid binding, protein phosphatase inhibitor activity, and protein serine/threonine kinase activity. The syntenic regions (at least five collinear homologous genes) across *P. fortunei*, *Olea europaea*, *M. guttatus*, *Tectona grandis,* and *Sesamum indicum* were used to investigate the *P. fortunei* whole-genome duplication (WGD) (Figure 5a). Based on the transversion substitutions at fourfold degenerated sites of collinear gene pairs, except for a core eudicot common hexaploidy event that occurred 115–130 million years ago, another WGD event that may have contributed to the divergence of *P. fortunei* and *T. grandis* was identified.

The divergence times of *P*. *fortunei* from the other 14 plants indicated that *P*. *fortunei* diverged from *T*. *grandis* approximately 38.8 (33.3–45.1) million years ago (Figure 5b), consistent with the WGD analysis results. Five expanded gene families (57 genes) and 79 contracted gene families (224 genes) in *P. fortunei* were, respectively, enriched in the defense response and proteolysis biological processes (Figure 5b). The GO classification of positively selected genes was enriched in the biological processes such as pseudouridine synthesis, Arp2/3 complex-mediated actin nucleation, and the molecular functions such as intramolecular transferase activity, pseudouridine synthase activity, isomerase activity, glutathione synthase activity, etc.

### 2.3. Whole-Genome Resequencing

The numbers of high-quality (HQ) clean reads from hybrid parents and F1 individuals were 42,751,840, 61,694,482, and 41,718,532–100,037,888, respectively, which finally yielded 971.45 Gb of HQ clean data with an average Q20 ratio of 97.13%, Q30 of 91.92%, and GC content of 36.73%. Using the above *P. fortunei* genome as a reference, the results of the BWA comparison showed that the average mapping number and proportion of total clean reads in the hybrid parents and F1 offspring were 5,899,863,136 and 90.55%, respectively. A total of 5,060,105 and 1,485,269 markers (SNPs and inDels) were discovered using the GATK v4.0 in the hybrid parents, respectively, and the average of 3,343,448 markers were determined in F1 progeny. Among these, the numbers of SNPs in the hybrid parents were 4,556,154 and 1,338,102, respectively, and their heterozygosis SNP rates were 81.38% and 63.71%, respectively. The average heterozygotic SNP rate of F1 individuals was 78.31%.

### 2.4. Genetic Map Construction

**Figure 6:**
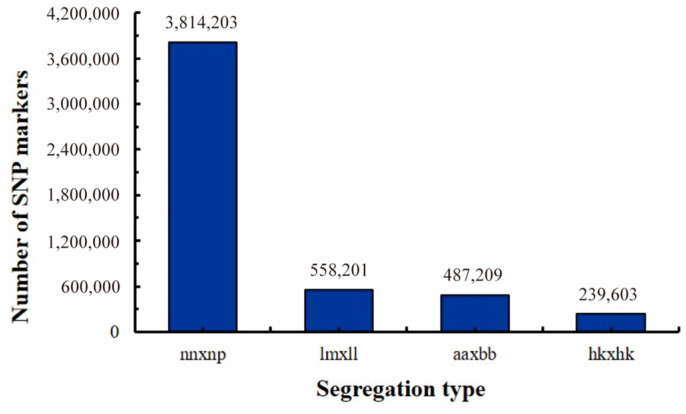
The segregation types of polymorphic SNP markers and high-density genetic map.

The separation types were determined according to the genotype of the hybrid parents; then, the hybrid F1 progenies were genotyped. A total of 5,099,216 polymorphic loci were found, among which 4,612,007 were segregation types nn × np, lm × ll, and hk × hk, accounting for 90.45% of the total. Thus, the three marker segregation types of nn × np (3,814,203), lm × ll (558,201), and hk × hk (239,603) were used on the genotype of the F1 population to construct genetic maps (Figure 6).

**Figure 7:**
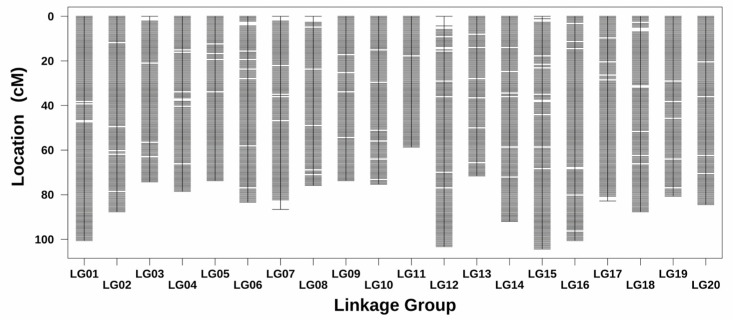
The high-density genetic map.

After statistical analysis, testing, and filtering, a total of 3,314,251 markers were finally obtained for further linkage analysis. After removing abnormal samples, a total of 93 samples were used for subsequent analysis. The marker types of lm × ll and hk × hk were used for constructing the male map, while nn × np and hk × hk were used to construct the female map; then, the integrated map was constructed by merging these two parent maps (Figure 7). The merged genetic map of 20 linked groups (LGs) contained 2993 bin markers, and the total genetic distance was 1675.14 cm. The average genetic distance between different markers was 0.56 cm, and the average number of SNPs and bins of a single LG were 165,639 and 150, respectively (Table 3). The average genetic distance of the 20 LGs was 83.76 cm, among which the smallest and largest were LG11 (58.61 cm, 109 bin markers) and LG15 (104.32 cm, 183 bin markers), respectively. Among these markers, 2972 gaps were detected, and no gaps were greater than 5 cm. Of these, 2864 gaps (96.37%) were between 0.5 and 1 cm, 101 gaps were between 1 and 2 cm, 6 gaps were between 2 and 3 cm, and only 1 gap was larger than 4 cm. The largest length of the maximum gap was 4.344 cm, distributed in LG07. The smallest length of the maximum gap was 1.076 cm, distributed in LG02, LG05, LG09, LG10, LG11, LG13, LG14, LG19, and LG20, respectively.

### 2.5. Statistical Analysis of Phenotypic Traits

**Figure 8:**
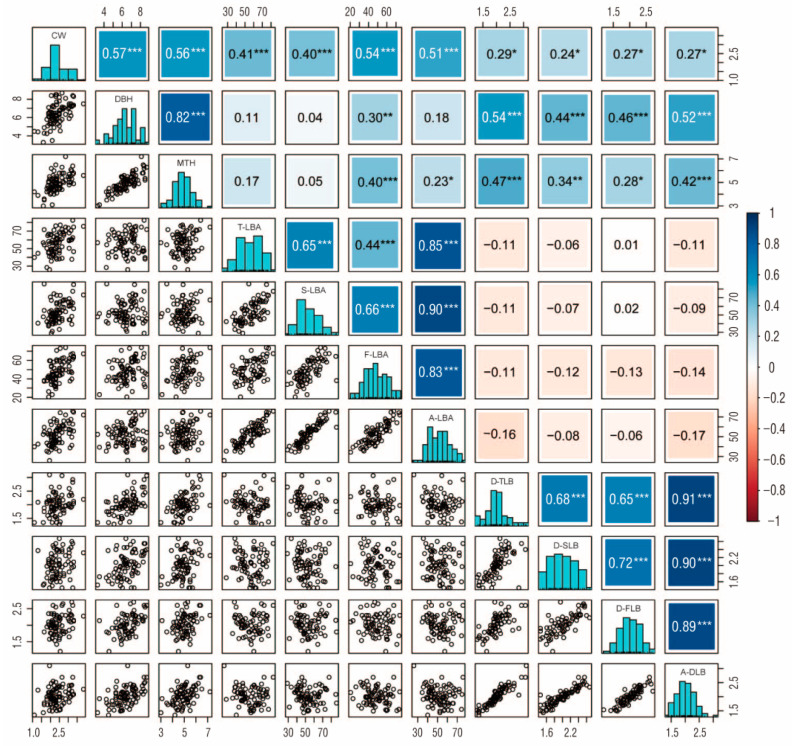
Pearson correlation coefficient among traits, trait distribution histograms, and correlation plots: *, **, *** represents *p* < 0.01, *p* < 0.001, and *p* < 0.0001, respectively. Positive and negative correlations for *p* < 0.0001 are shown in blue and orange, respectively.

The results of statistical analysis showed that the coefficients of variation in phenotypic traits ranged from 15.03% to 24.57% (Table 4), and the individual variation was large, showing obvious separation. Among them, the traits with the largest and smallest coefficients of variation were the first lateral branch angle (F-LBA) and main trunk height (MTH), respectively. The results also showed that most of the 11 phenotypic traits had normal distributions, indicating that most were quantitative traits whose inheritance was jointly controlled by multiple mini-effect genes (Table 4, Figure 8). The high, significant positive correlations were observed between the diameter at breast height (DBH) and MTH (0.82), between the average lateral branch angle (A-LBA) and F-LBA (0.83), the second lateral branch angle (S-LBA) (0.90), the third lateral branch angle (T-LBA) (0.85), between the average diameter of the lateral branch (A-DLB) and diameter of the first lateral branch (D-FLB) (0.89), the diameter of the second lateral branch (D-SLB) (0.90), and the diameter of the third lateral branch (D-TLB) (0.91). There were moderate significant positive correlations between D-FLB, D-SLB, and D-TLB (0.65–0.72), similar to those between F-LBA, S-LBA, and T-LBA (0.44–0.66). The DBH, MTH, F-LBA, S-LBA, T-LBA, and A-LBA showed moderate significant positive correlations with crown width (CW) (0.40–0.57). The D-FLB, D-SLB, D-TLB, and A-DLB also showed moderate significant positive correlations with DBH (0.44–0.54) (Figure 8).

### 2.6. QTL Location Analysis

**Figure 9:**
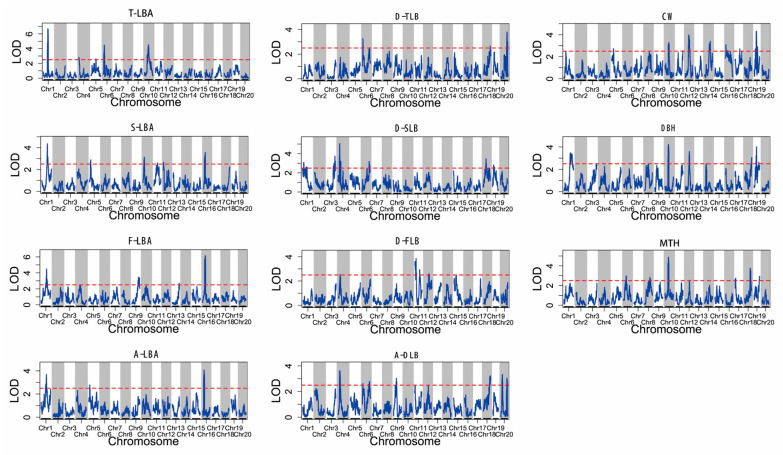
The 11 phenotypic traits related to QTL mapping among all linkage groups content: the red dashed lines represent LOD = 2.5.

Based on the integrated genetic map, the composite interval mapping (CIM) function of R/qtl was used for the complex interval mapping of each trait. A total of 73 QTLs were identified with logarithm of odds (LOD) values ranging from 2.50 to 6.68. Among these, the number of QTLs controlling CW, DBH, and D-SLB were the highest (11, 10, and 9, respectively), followed by A-DLB (8), MTH (7), S-LBA (6), D-FLB (6), T-LBA (5), F-LBA (5), A-LBA (3), and D-TLB (3) (Table 5). These QTLs were distributed on 18 linkage groups except LG2 and LG7, and the distribution of QTLs on each linkage group was uneven, among which the number of QTLs located in the LG1, LG6, LG11, and LG19 were relatively large, with 9, 6, 6, and 6, respectively. The LG13 and LG15 each contained only one QTL, while the LG3, LG8, and LG14 each contained two QTLs. Figure 9 shows the 11 phenotypic traits related to QTL mapping among all linkage groups content.

The 11 traits could be divided into three categories (I, II, and III). Categories I and II included MTH and DBH, respectively, while the third category (III) included the branching characters CW, F-LBA, S-LBA, T-LBA, A-LBA, D-FLB, D-SLB, D-TLB, and A-DLB. Seven QTLs were discovered for category I on chromosome LGs 6, 8, 10, 17, 18, and 19, and the percentage of phenotypic variation explained (PVE) for each QTL was different, ranging from 0.66% to 12.68%. There were two major QTLs with PVE > 10%, accounting for 28.57% of the total QTLs (7), and LOD values ranged from 2.62 to 4.88. We identified ten QTLs for category II on chromosome LGs 1, 8, 10, 12, 14, 18, and 19 with PVE by each QTL varying from 0.48% to 13.97%. There was only one major QTL on LG10 with PVE > 10% and LOD = 4.24. For category III, 56 QTLs were identified on chromosome LGs 1, 3, 4, 5, 6, 9, 10, 11, 12, 13, 14, 15, 16, 17, 18, 19, and 20 with PVEs ranging from 0.15% to 28.63%. Among these, there were 16 major QTLs with PVE > 10% (LOD = 2.59 -6.88) and 4 major QTLs with PVE > 15% (LOD = 3.69–6.68). The two QTLs located in LG1 and LG6 had the highest PVE of 28.63% and 15.48% for T-LBA, respectively, followed closely by the QTL on LG19 with a PVE of 16.49% for CW and the QTL on LG1 with a PVE of 15.75% for A-LBA. The QTLs with the highest average PVEs were found for T-LBA (13.52%), A-LBA (11.41%), and D-TLB (9.32%).

All the genes in the 73 QTL intervals were annotated functionally based on the assembled *P. fortunei* genome; classification and KEGG pathway enrichment analyses were further carried out. The results showed that the two major QTLs (PVE ≥ 10%) of MTH contained 43 functional genes, which were enriched in 49 KEGG pathways, of which two pathways were significantly enriched (*p*-value < 0.05). Eighteen functional genes were identified in only one major QTL (PVE ≥ 10%) of DBH, which were enriched in twenty-four KEGG pathways, and of which six pathways were significantly enriched (*p*-value < 0.05). Nine KEGG pathways were shared by F-LBA, S-LBA, T-LBA, A-LBA, and CW, namely the amino sugar and nucleotide sugar metabolism (ko00520), the biosynthesis of secondary metabolites (ko01110), metabolic pathways (ko01100), phenylpropanoid biosynthesis (ko00940), the phosphatidylinositol signaling system (ko04070), plant–pathogen interaction (ko04626), protein processing in endoplasmic reticulum (ko04141), ribosome (ko03010), and starch and sucrose metabolism (ko00500). The D-FLB, D-SLB, D-TLB, and A-DLB shared two KEGG pathways, namely the biosynthesis of secondary metabolites (ko01110) and metabolic pathways (ko01100), which were also shared by nine branch-related traits.

The A-LBA was used to represent the lateral branch angle of the plant on account of its highly significant positive correlations with F-LBA, S-LBA, and T-LBA. Similar to the lateral branch angle, the A-DLB was used to represent the diameter of the lateral branch of the plant. Therefore, MTH, DBH, CW, A-LBA, and A-DLB were selected as representative traits; the three KEGG pathways with the smallest *p*-values and the candidate genes of each trait are listed in Table S1, respectively. The results showed that A-LBA and MTH shared one KEGG metabolic pathway, namely cutin, suberine, and wax biosynthesis (ko00073)), and also shared one candidate gene, namely the alcohol-forming fatty acyl-CoA reductase (*FAR*, [EC:1.2.1.84]). Moreover, the A-LBA and DBH shared one KEGG metabolic pathway, namely Zeatin biosynthesis (ko00908), and also shared two candidate genes, namely cytokinin dehydrogenase (CKX, [EC:1.5.99.12]) and cis-zeatin O-glucosyltransferase (CISZOG, [EC:2.4.1.215]).

## 3. Discussion

High-quality genome assembly has important guiding significance for the genetic breeding of *Paulownia*. In this research, the *P. fortunei* genome was constructed via hybrid assembly of Illumina, PacBio, and chromatin interaction mapping data, with a size of approximately 476.82 Mb, 0.52% heterozygosity, contig N50s of 7.81 Mb, and scaffold N50s of 21.81 Mb. Compared to the first *P. fortunei* genome reported in 2021 with a size of 511.6 Mb, a heterozygosity of 2.03%, and an N50 length of 852.4 kb, this assembly had greatly reduced heterozygosity and increased N50 length [7], therefore greatly enriching the genomic data of the *P. fortunei* species. Previous studies on *Paulownia* classification have mostly been based on morphological features, chloroplast genome information, or partial nuclear gene information, all of which have certain limitations, so there was no definite conclusion [30,31,32]. In Angiosperm Phylogeny Group (APG) III, Orobanchaceae and Phrymaceae form a clade, with Paulowniaceae and Lamiaceae being successive sister groups [31]. In APG IV, Phrymaceae is sister to a clade comprising the Paulowniaceae and Orobanchaceae, followed by the Lamiaceae [30,32]. In this study, the phylogenetic tree based on complete genome data showed that Phrymaceae was sister to a clade composed of Paulowniaceae and Labiatae, which was slightly different from other results [7]. It was speculated that this may have been due to the different species selected when building the phylogenetic tree, the limited number of species, and other reasons. Therefore, with the release of genome data for more species in the future, it will be necessary to reassess the phylogenetic status of Paulowniaceae in a timely and accurate manner.

The number of molecular markers plays a decisive role in the quality of the genetic linkage map [33]. The early genetic maps have mostly been constructed using restriction fragment length polymorphisms, SSRs, random amplified polymorphic DNA, and other markers, but these processes have been cumbersome and time-consuming, the marker density has been low, and the results have not provided accurate and complete QTL number and location information [34,35,36]. The rapid development and application of high-throughput sequencing technology have greatly improved the quality of these maps and promoted the development of high-density genetic maps. Particularly, whole-genome resequencing technology can quickly obtain markers with a wider, more uniform distribution and a larger number of genomes, and the genetic map constructed is accordingly more dense and of higher quality, which is conducive to the accurate positioning of quantitative traits in the future. At present, such methods have been widely used to construct high-density genetic maps of woody plants such as poplar [37], eucalyptus [38]^,^ and *Camellia sinensis* [39]. Genetic maps constructed based on the segmentation bin strategy can provide accurate physical locations due to their high quality. Major QTLs related to important economic traits have successfully been identified in many plants, such as loquat [40], *capsicum* [41]^,^ and wheat [42]. In this study, based on the whole-genome resequencing of the hybrid parents and 93 F1 progenies, a high-quality *Paulownia* genetic map containing 2993 bin (3,312,780 SNPs) markers was constructed based on the segmentation bin strategy. The total map distance was 1,675.14 cm, and the average genetic distance between different markers was 0.56 cm. The number of markers and the marker density of the above map were both higher than those of the first *Paulownia* genetic map constructed using the RAD-seq method in 2018 [43] and more beneficial for the identification of QTL in adjacent positions.

Of course, the QTL mapping results and accuracy of different phenotypic traits differ in different plants, mainly depending on the size of the mapping population, the quality of the map, the distribution characteristics of the traits, and so forth. This study had some limitations because the population size was relatively small (93), which may have resulted in broken linkage groups and inaccurate site information [44]. However, there have also been reports of the successful use of high-density genetic maps constructed with small-scale populations for QTL localization [45]. The results of correlation analysis revealed a high or moderate, significant, positive correlation between four branch angle traits, similar to those between four branch diameter traits. The DBH, MTH, F-LBA, S-LBA, T-LBA, and A-LBA showed moderate significant positive correlations with CW. Based on these observations, we carried out QTL mapping studies on important phenotypic traits such as DBH, MTH, and branching characteristics of *Paulownia* and identified 73 QTLs. Seven of these were related to MTH, ten to DBH, and fifty-six to branching-related traits. The PVEs of the 73 QTLs ranged from 0.15% to 28.63% and included four major QTLs with PVE > 15%, all related to branching properties. The QTLs controlling shoot branching were detected in Chinese cabbage, with PVEs varying from 5.44% to 22.18% [12]. Seventy-one QTLs for branching-related characters were identified in Schima superba, with PVEs ranging from 12.8% to 22.8% [46]. Thirty QTLs for leaf traits and needling length were discovered in *Ziziphus jujube*, with PVE ranging from 13.3% to 29.9% [47].

In this study, the merged genetic map constructed based on bin markers can effectively combine markers of the same genotype to avoid marker redundancy. Moreover, QTL localization and specific screening methods can effectively avoid candidate marker redundancy caused by linkage disequilibrium and improve localization accuracy. The *FAR* gene shared by A-LBA and MTH has been shown to be associated with suberin biosynthesis [48,49]. In addition, the A-LBA and DBH shared two candidate genes, namely *CKX* and *CISZOG*, respectively. Previous research has shown that *CKX* participates in various physiological processes of plants, such as CK catabolism metabolism, leaf angle, and abiotic stress resistance [50,51], which is very important for plant growth and development. The transgenic tobacco plants harboring the *ZOG1* gene showed characteristics indicative of cytokinin deficiency; meanwhile, the exception was the occasional branching of transformed plants [52]. Although this study provided a large number of candidate genes for important phenotypic traits, the key gene screening, expression analysis, function verification, and genotype × environment association analysis for regulating different traits need to be further explored. The most important question is, through what means to achieve the purpose of *Paulownia*-directed genetic improvement (gene editing or recurrent backcrossing) is also worth exploring.

## 4. Materials and Methods

### 4.1. Genome Sequencing, Assembly, and Annotation

*P*. *fortunei* samples were collected from Jiujiang City, Jiangxi Province, China (115°45′0″ E, 29°19′48″ N). The sampled specimen was 8 years old, with a diameter at breast height and tree height of 44.30 cm and 15.10 m, respectively. The DNA was extracted from young leaves for genome sequencing. The short cleaned reads from Illumina HiSeq X Ten were used for a genome survey, base level correction after assembly, and Hi-C library construction; the long cleaned reads from the PacBio SEQUEL platforms were used for genome assembly, and the Hi-C library was constructed according to the methods of Eitan et al. [53]. After extraction and quality testing of RNA from the stem segment, leaf blade, petiole, petal, and sepal, RNA sequencing libraries were constructed and sequenced on the Illumina HiSeq PE150 platform. *K-mer* frequency analysis was used to estimate the genome characteristics of *P*. *fortunei*. The genome size of *P*. *fortunei* was calculated based on *K-mer* (*k* = 17) statistics using the modified Lander–Waterman algorithm.

De novo assembly of the PacBio long reads was performed using the CANU pipeline run with the default parameters. Pilon was used to perform error correction based on the Illumina sequences. Then, Hi-C sequencing data were aligned to the assembled scaffolds using the Burrows–Wheeler aligner, and the scaffolds were clustered onto chromosomes using LACHESIS. The BUSCO and CEGMA were used to assess the completeness of the assembled *P*. *fortunei* genome, respectively. The genome accuracy was evaluated via the BWA. The RepeatModeler, LTR_FINDER, and RepeatScout were used to build a de novo repeat library. For the homology-based approach, we used the RepeatMasker against the Repbase TE library and the RepeatProteinMask against the TE protein database. Tandem repeats in the genome were detected using TRF.

For de novo gene structure predictions, Augustus, GlimmerHMM, SNAP, Geneid, and GENSCAN analyses were performed on the repeat-masked genome. Homologous species, including *Arabidopsis thaliana*, *Populus trichocarpa*, *Rhodiola crenulata*, *Salvia miltiorrhiza*, *Solanum lycopersicum*, *Eucalyptus grandis*, and *Amborella trichopoda,* were used for annotation and referencing. Transcriptome read assemblies were generated using Trinity, and annotations were generated using HISAT, StringTie, and a program to assemble spliced alignment. Functional annotation of protein-coding genes was performed using BLASTP against the SwissProt and non-redundant protein sequence databases. InterProScan and HMMER were applied to annotate the protein domains by searching the InterPro and Pfam databases, respectively. GO terms were obtained from the corresponding InterPro or Pfam entry, and the pathways were identified using BLAST against the KEGG database. The tRNA genes were identified using tRNAscan-SE. The rRNA fragments were predicted via alignment to rRNA sequences using BlastN. The miRNA and snRNA genes were predicted using Infernal against the Rfam database.

### 4.2. Comparative Genome Analysis

Comparative genome analysis was performed to identify orthologous gene families among the 15 sequenced species, namely, *P. fortunei*, *A. trichopoda*, *Oryza sativa*, *A. thaliana*, *P. trichocarpa*, *Salix purpurea*, *E. grandis*, *Fraxinus excelsior*, *O. europaea*, *M. guttatus*, *T. grandis*, *S. indicum*, *S. lycopersicum*, *Actinidia chinensis*, and *Spinacia oleracea*. For all-against-all protein BLAST analyses, proteins of fewer than 50 amino acids or containing the termination codon inside were filtered out, while the longest protein among alternative splicing variants was retained. BLASTP (E-value of1 × 10^−5^) was used to blast the filtered proteins and cluster them into orthologous groups using OrthoMCL (inflation parameter = 1.5). The interactive graphs (p-values associated with GO categories) of these specific genes based on the GO enrichment analysis results were drawn using REViGO with the whole UniProt database as the GO term size and SimRel as the semantic similarity measure.

The fourfold degenerate synonymous site (4DTv) was used for the WGD event assay. One protein per species in a cluster was clustered into single-copy orthologues, and a phylogenetic tree was generated using the maximum likelihood method with RAxML. The divergence time of each species was estimated via MCMCtree in phylogenetic analysis using the maximum likelihood, and the time correction points were taken from the TimeTree website. The gene family expansion/contraction analysis was conducted using CAFE. The branch-site model in PAML was used to detect whether the gene family was positively selected in a species based on the *Ka*/*Ks* ratio (*q*-value < 0.05).

### 4.3. Whole-Genome Resequencing and Genetic Map Construction

In April 2019, the hybridization of *P. catalpifolia* and *P. fortunei* was carried out in Mengzhou, Jiaozuo City, Henan Province (112.71° E, 34.86° N), and then, the seedlings with hybrid seeds were bred in 2020. In the spring of 2021, a total of 150 individual F1 plants were randomly selected and planted with consistent tending measures in Zhongxiang, Jingmen City, Hubei Province (112.73° E, 31.17° N). After removing the abnormal individuals, such as those with severe disease or pest infestation, wind breakage, and other accidental injuries, the remaining 98 individuals were used to construct the genetic map. In September 2022, the young leaves of female parents, male parents, and 98 individual F1 plants were collected to extract genomic DNA using the CTAB method [54]. After a series of operations (such as DNA fragmentation, terminal repair, adding poly (A) tail and adapters, and so forth), the qualified DNAs were used to construct the sequencing libraries. The qualified libraries were finally sequenced via double-terminal PE150 on the Illumina Hiseq X10 platform ((Illumina, San Diego, CA, USA) with a sequencing depth of 10×. The software BWA v0.7.17 was used to compare the filtered, high-quality, clean data to the reference genome of *P. fortunei*. After the mutation sites were detected, corrected, integrated, filtered, and so forth, the high-quality population markers obtained were classified into corresponding linkage groups and sorted according to the physical location information of the markers. The software Lep-MAP3 v0.5 [55,56] was used to calculate the genetic distance between markers and the map distance of each chromosome. The marker with the highest integrity (lowest deletion rate) was selected as the bin marker in each genetic position. If there were multiple markers in the highest integrity condition, the first marker was selected as the representative. The physical interval of a bin is defined as the physical position corresponding to the start marker of the genetic position to the physical position corresponding to the first marker of the next genetic position minus 1. The genetic position of the bin still uses the genetic location calculated using all of the above markers. The male and female genetic maps were constructed based on bin markers, respectively, and then, the integrated genetic map was constructed by merging these two parent maps. The integrated genetic map quality was evaluated by comprehensive evaluation of the genetic length, the number of large gaps, the graphic genotype, the graphic linkage strength, and so forth.

### 4.4. Analysis of Phenotypic Traits and QTL Location

The DBH and diameters of the lateral branch (DLBs) were measured with a tape measure. The CW was measured with a meter stick and divided into two directions: north and south, east and west. The MTH was measured with a flower pole. The lateral branch angles (LBAs) were measured with an angle ruler with a digital display. The DLBs and LBAs were measured in three rounds from top to bottom, namely the first, second, and third rounds (D-FLB/F-LBA, D-SLB/S-LBA, and D-TLB/S-LBA), and the average DLB and LBA (A-DLB and A-LBA) were taken as the average of the three rounds. The degree of variation in phenotypic traits among individuals was analyzed using the coefficient of variation. The skewness and kurtosis were used to evaluate the distribution of phenotypic data for each trait. Pearson’s correlation coefficient was used to analyze the correlation between different traits. The constructed genetic map and phenotypic data were combined to determine the locations of QTLs using CIM in software R/qtl v1.42-8. The scanning step was 1 cm, and LOD = 2.5 was used as the threshold value to screen QTLs. The significant intervals were screened according to thresholds; the vertex of the significant interval was taken as the peak of the QTL, multiple QTLs with peak distances less than 10 cm were merged into one, and the most significant peak was taken as the combined peak. Within 10 cm at both ends of the peak, the interval included by reducing 1.5 LOD values was used as the confidence interval for this QTL. If the peak position is extended 10 cm to one side, and the LOD value still does not decrease by 1.5, then the 10 cm position is taken as the QTL boundary. The functional annotation and KEGG pathways enrichment analysis of genes in the QTL interval were further performed.

## 5. Conclusions

In conclusion, the present study constructs high-quality chromosomal genome sequences of *P. fortunei*, with a size of 476.82 Mb, heterozygosity of 0.52%, and contig and scaffold N50s of 7.81 Mb and 21.81 Mb, respectively. Twenty scaffolds with a total length of 437.72 Mb were assembled into twenty pseudochromosomes. The *P. fortunei* genome can lay a solid foundation for genomic structure and evolution, genetic diversity, and phylogeny. Further comparative genomics analysis preliminarily showed that the split of *P. fortunei* with *T. grandis* likely occurred 38.8 (33.3–45.1) million years ago. The merged genetic map of 20 linkage groups was constructed using whole-genome resequencing, with 2993 bin markers and a total length of 1675.14 cm. In total, 73 QTLs for important phenotypic traits were identified, including 19 major QTLs with phenotypic variation explained ≥ 10%. The QTLs above and candidate genes identified in the present study will be the main focus of *Paulownia* breeders for fine mapping and gene cloning of *Paulownia* varieties with improved growth and crown type.

## Figures and Tables

**Table 1:** Summary of the final genome assembly of *P. fortunei*.

Term	Length		Number	
	Contig (bp)	Scaffold (bp)	Contig	Scaffold
Total	476,781,199	476,819,499	807	424
Max	14,548,517	28,641,687	-	-
Number ≥ 2000	-	-	799	417
N50	7,808,517	21,809,624	23	10
N60	6,515,000	20,891,980	29	12
N70	5,045,907	20,069,062	38	15
N80	2,390,000	17,814,499	50	17
N90	153,543	16,229,908	171	20

**Table 2:** The statistical results of gene function annotation of the *P. fortunei* genome.

Database Name	Gene Number	Percent (%)
Total	26,903	-
Swissprot	21,905	81.42
Nr	25,951	96.46
KEGG	20,911	77.73
InterPro	25,273	93.94
GO	15,648	58.16
Pfam	21,309	79.21
Annotated	26,008	96.67
Unannotated	895	3.33

**Table 3:** The linkage group statistics of the genetic map.

Linkage Group	Number of Markers ^a^	Length of Male Map (cm) ^b^	Length of Female Map (cm) ^b^	Length of Integrated Map (cm) ^b^	Average Distance (cm) ^c^	Maximum Gap (cm) ^d^
LG01	184	86.04	115.07	100.56	0.55	1.62
LG02	159	66.68	108.62	87.65	0.55	1.08
LG03	133	67.76	80.66	74.21	0.56	2.16
LG04	140	62.38	94.65	78.51	0.56	1.62
LG05	134	73.13	74.21	73.67	0.55	1.08
LG06	147	74.21	92.50	83.35	0.57	2.16
LG07	148	75.38	97.87	86.62	0.59	4.34
LG08	133	65.63	86.04	75.83	0.57	2.70
LG09	134	62.38	84.96	73.67	0.55	1.08
LG10	135	59.15	91.41	75.28	0.56	1.08
LG11	109	54.85	62.38	58.61	0.54	1.08
LG12	176	89.28	117.25	103.26	0.59	4.31
LG13	128	59.15	83.89	71.52	0.56	1.08
LG14	166	78.51	105.40	91.95	0.55	1.08
LG15	183	88.19	120.45	104.32	0.57	1.62
LG16	181	78.51	122.60	100.56	0.56	1.62
LG17	148	67.76	97.87	82.82	0.56	2.16
LG18	155	74.22	101.09	87.66	0.57	2.16
LG19	146	72.06	89.26	80.66	0.55	1.08
LG20	154	73.13	95.71	84.42	0.55	1.08
Total	2993	1428.40	1921.88	1675.14	0.56	4.34

Note: ^a^: The number of bin markers; ^b^: The genetic distance of linkage groups in male, female, and integrated maps, respectively. ^c^: The average genetic distance between markers. ^d^: The length of the maximum gap.

**Table 4:** The variation analysis of 11 phenotypic traits.

Traits	Sample Number	Average	Range of Variation	Variation Coefficient	Skew	Kurtosis
MTH	84	4.96	3~7.2	15.03	0.08	0.46
DBH	84	6.39	3.3~8.68	18.47	−0.22	−0.28
CW	84	2.39	1.1~3.85	20.85	0.34	0.09
F-LBA	76	48.34	20.5~74	24.57	0.05	−0.57
S-LBA	79	53.41	29~86.05	22.69	0.52	−0.19
T-LBA	79	55.96	26~82.55	22.15	−0.09	−0.74
A-LBA	84	52.03	26~75.85	20.26	0.21	−0.51
D-FLB	72	2.03	1.21~2.7	15.20	−0.01	−0.32
D-SLB	77	2.01	1.45~2.68	15.75	0.09	−0.98
D-TLB	77	2.03	1.29~3.1	19.02	0.46	0.44
A-DLB	84	2.03	1.35~3.1	15.83	0.33	0.31

Note: MTH: main trunk height; DBH: diameter at breast height; CW: crown width; F-LBA: the first lateral branch angle; S-LBA: the second lateral branch angle; T-LBA: the third lateral branch angle; A-LBA: the average lateral branch angle; D-FLB: diameter of the first lateral branch; D-SLB: diameter of the second lateral branch; D-TLB: diameter of the third lateral branch; A-DLB: the average diameter of the lateral branch.

**Table 5:** The QTL identification results of 11 phenotypic traits.

Traits	Number of QTLs	Linkage Groups	Range of PVE (%)	Range of LOD Value
MTH	7	6, 8, 10, 17, 18, 19	0.66–12.68	2.62–4.88
DBH	10	1, 8, 10, 12, 14, 18, 19	0.48–13.97	2.50–4.24
CW	11	5, 10, 11, 12, 14, 16, 17, 19	0.66–16.49	2.54–4.32
F-LBA	5	1, 9, 13, 16	0.15–14.55	2.69–6.15
S-LBA	6	1, 5, 10, 11, 12, 16	2.32–12.45	2.61–4.35
T-LBA	5	1, 4, 5, 6, 10	4.24–28.63	2.60–6.68
A-LBA	3	1, 5, 16	4.76–15.75	2.78–4.07
D-FLB	6	4, 11, 12, 15	1.88–10.13	2.50–3.86
D-SLB	9	1, 3, 4, 6, 18, 19	1.81–14.32	2.60–5.04
D-TLB	3	6, 18, 20	5.99–11.00	2.66–3.80
A-DLB	8	4, 6, 9, 11, 18, 20	2.05–12.05	2.50–3.63

Note: the annotations of trait names were the same as in Table 4; PVE: phenotypic variation explained; LOD: logarithm of odds.

## Data Availability

The raw sequencing data of the *P. fortunei* whole genome are available in the NCBI (SUBID: SUB9191782; BioProject: PRJNA689913).

## Supplementary Materials

The following supporting information can be downloaded at https://www.mdpi.com/article/10.3390/ijms242115647/s1.
