# Supplementary material for: Construction of a High-Density Paulownia Genetic Map and QTL Mapping of Important Phenotypic Traits Based on Genome Assembly and Whole-Genome Resequencing
Source: Int J Mol Sci. 2023 Oct 27;24(21):15647. doi: 10.3390/ijms242115647 (PMC10647314; doi:10.3390/ijms242115647)
Supplement: Supplementary file 1 [file ijms-24-15647-s001.zip › ijms-2652513-supplementary.pdf]

Table S1. The KEGG pathway enrichment analysis results and candidate gene profiles.

Traits	NO.	Pathway	Pathway ID	K_id	candidate genes	K_id description
MTH	1	Valine, leucine and isoleucine biosynthesis	ko00290	K00826	evm.TU.Chr10.726(BCAT2)	E2.6.1.42, ilvE; branched-chain amino acid aminotransferase [EC:2.6.1.42]
				K01649	evm.TU.Chr6.708(IPMSB)	leuA, IMS; 2-isopropylmalate synthase [EC:2.3.3.13]
				K01649	evm.TU.Chr6.709(IPMSA)	leuA, IMS; 2-isopropylmalate synthase [EC:2.3.3.13]
				K01649	evm.TU.Chr6.710(IPMSB)	leuA, IMS; 2-isopropylmalate synthase [EC:2.3.3.13]
				K01649	evm.TU.Chr6.712(IPMSA)	leuA, IMS; 2-isopropylmalate synthase [EC:2.3.3.13]
				K01649	evm.TU.Chr6.713(IPMSB))	leuA, IMS; 2-isopropylmalate synthase [EC:2.3.3.13]
				K01687	evm.TU.Chr6.486(DHAD)	ilvD; dihydroxy-acid dehydratase [EC:4.2.1.9]
				K01703	evm.TU.Chr6.423(IIL1)	leuC, IPMI-L; 3-isopropylmalate/(R)-2-methylmalate dehydratase large subunit [EC:4.2.1.33 4.2.1.35]
	2	2-Oxocarboxylic acid metabolism	ko01210	K00826	evm.TU.Chr10.726(BCAT2)	E2.6.1.42, ilvE; branched-chain amino acid aminotransferase [EC:2.6.1.42]
				K00928	evm.TU.Chr19.861(AK1)	lysC; aspartate kinase [EC:2.7.2.4]
				K01649	evm.TU.Chr6.708(IPMSB)	leuA, IMS; 2-isopropylmalate synthase [EC:2.3.3.13]
				K01649	evm.TU.Chr6.709(IPMSA)	leuA, IMS; 2-isopropylmalate synthase [EC:2.3.3.13]
				K01649	evm.TU.Chr6.710(IPMSB)	leuA, IMS; 2-isopropylmalate synthase [EC:2.3.3.13]
				K01649	evm.TU.Chr6.712(IPMSA)	leuA, IMS; 2-isopropylmalate synthase [EC:2.3.3.13]
				K01649	evm.TU.Chr6.713(IPMSB))	leuA, IMS; 2-isopropylmalate synthase [EC:2.3.3.13]
				K01687	evm.TU.Chr6.486(DHAD)	ilvD; dihydroxy-acid dehydratase [EC:4.2.1.9]
				K01703	evm.TU.Chr6.423(IIL1)	leuC, IPMI-L; 3-isopropylmalate/(R)-2-methylmalate dehydratase large subunit [EC:4.2.1.33 4.2.1.35]
				K14682	evm.TU.Chr10.696_evm.TU.Chr10.697(NAGS1)	argAB; amino-acid N-acetyltransferase [EC:2.3.1.1]
	3	Cutin, suberine and wax biosynthesis	ko00073	K13356	evm.TU.Chr6.803(FAR2)	FAR; alcohol-forming fatty acyl-CoA reductase [EC:1.2.1.84]
				K13356	evm.TU.Chr6.804(FAR2)	FAR; alcohol-forming fatty acyl-CoA reductase [EC:1.2.1.84]
				K13356	evm.TU.Chr6.805(--)	FAR; alcohol-forming fatty acyl-CoA reductase [EC:1.2.1.84]
				K13356	evm.TU.Chr6.806(FAR2)	FAR; alcohol-forming fatty acyl-CoA reductase [EC:1.2.1.84]
				K13356	evm.TU.Chr6.807(--)	FAR; alcohol-forming fatty acyl-CoA reductase [EC:1.2.1.84]
				K13356	evm.TU.Chr6.808_evm.TU.Chr6.809(FAR2)	FAR; alcohol-forming fatty acyl-CoA reductase [EC:1.2.1.84]
				K17991	evm.TU.Chr6.404(SOP1)	PXG; peroxygenase [EC:1.11.2.3]
				K20769	evm.TU.Chr8.1279(CYP94A2)	CYP94A5; fatty acid omega-hydroxylase [EC:1.14.-.-]

DBH	1	Zeatin biosynthesis	ko00908	K00279	evm.TU.Chr1.1829(CKX1)	CKX; cytokinin dehydrogenase [EC:1.5.99.12]
				K13495	evm.TU.Chr1.1845(ZOX1)	CISZOG; cis-zeatin O-glucosyltransferase [EC:2.4.1.215]
				K13495	evm.TU.Chr1.1846(ZOX1)	CISZOG; cis-zeatin O-glucosyltransferase [EC:2.4.1.215]
				K13495	evm.TU.Chr1.1847(ZOX1)	CISZOG; cis-zeatin O-glucosyltransferase [EC:2.4.1.215]
				K13495	evm.TU.Chr1.1849(--)	CISZOG; cis-zeatin O-glucosyltransferase [EC:2.4.1.215]
				K13495	evm.TU.Chr1.1850(ZOX1)	CISZOG; cis-zeatin O-glucosyltransferase [EC:2.4.1.215]
				K13495	evm.TU.Chr1.1851(ZOX1)	CISZOG; cis-zeatin O-glucosyltransferase [EC:2.4.1.215]
				K13495	evm.TU.Chr1.1852(ZOX1)	CISZOG; cis-zeatin O-glucosyltransferase [EC:2.4.1.215]
				K13495	evm.TU.Chr1.1853(ZOX1))	CISZOG; cis-zeatin O-glucosyltransferase [EC:2.4.1.215]
	2	Linoleic acid metabolism	ko00591	K00454	evm.TU.Chr19.731(LOX6)	LOX2S; lipoxygenase [EC:1.13.11.12]
				K00454	evm.TU.Chr8.1067(LOX3.1)	LOX2S; lipoxygenase [EC:1.13.11.12]
				K00454	evm.TU.Chr8.1108(LOX2.1)	LOX2S; lipoxygenase [EC:1.13.11.12]
				K14674	evm.TU.Chr1.1805(SDP1)	TGL4; TAG lipase / steryl ester hydrolase / phospholipase A2 / LPA acyltransferase [EC:3.1.1.3 3.1.1.13 3.1.1.4 2.3.1.51]
	3	Synthesis and degradation of ketone bodies	ko00072	K01640	evm.TU.Chr1.1744(HMGCL)	E4.1.3.4, HMGCL, hmgL; hydroxymethylglutaryl-CoA lyase [EC:4.1.3.4]
				K01640	evm.TU.Chr8.960(HMGCL)	E4.1.3.4, HMGCL, hmgL; hydroxymethylglutaryl-CoA lyase [EC:4.1.3.4]
				K01641	evm.TU.Chr14.474(HMGS)	E2.3.3.10; hydroxymethylglutaryl-CoA synthase [EC:2.3.3.10]
CW	1	Nitrogen metabolism	ko00910	K00261	evm.TU.Chr12.829(GDHA)	GLUD1_2, gdhA; glutamate dehydrogenase (NAD(P)+) [EC:1.4.1.3]
				K00284	evm.TU.Chr5.187(FdGOGAT)	GLU, gltS; glutamate synthase (ferredoxin) [EC:1.4.7.1]
				K01673	evm.TU.Chr16.162(BCA1)	cynT, can; carbonic anhydrase [EC:4.2.1.1]
				K01673	evm.TU.Chr19.577(BCA2)	cynT, can; carbonic anhydrase [EC:4.2.1.1]
				K01674	evm.TU.Chr17.720(ACA7)	cah; carbonic anhydrase [EC:4.2.1.1]
				K02575	evm.TU.Chr16.144(NRT2.7)	NRT, narK, nrtP, nasA; MFS transporter, NNP family, nitrate/nitrite transporter
				K02575	evm.TU.Chr17.707(NRT2.4)	NRT, narK, nrtP, nasA; MFS transporter, NNP family, nitrate/nitrite transporter
				K02575	evm.TU.Chr17.708(NRT2.1)	NRT, narK, nrtP, nasA; MFS transporter, NNP family, nitrate/nitrite transporter
				K02575	evm.TU.Chr17.709(NRT2.1)	NRT, narK, nrtP, nasA; MFS transporter, NNP family, nitrate/nitrite transporter
	2	Ubiquitin mediated proteolysis	ko04120	K03178	evm.TU.Chr17.1043(UBA1)	UBE1, UBA1; ubiquitin-activating enzyme E1 [EC:6.2.1.45]
				K03349	evm.TU.Chr19.374(APC2)	APC2; anaphase-promoting complex subunit 2
				K03363	evm.TU.Chr12.806(CDC20-1)	CDC20; cell division cycle 20, cofactor of APC complex
				K03363	evm.TU.Chr12.807(--)	CDC20; cell division cycle 20, cofactor of APC complex

				K03869	evm.TU.Chr19.417(CUL3A)	CUL3; cullin 3
				K03872	evm.TU.Chr17.877(elc1)	ELOC, TCEB1; elongin-C
				K04706	evm.TU.Chr17.1086(At1g65750)	PIAS1; E3 SUMO-protein ligase PIAS1 [EC:2.3.2.-]
				K06688	evm.TU.Chr17.757(UBC20)	UBE2C, UBC11; ubiquitin-conjugating enzyme E2 C [EC:2.3.2.23]
				K10140	evm.TU.Chr17.1013(DDB2)	DDB2; DNA damage-binding protein 2
				K10573	evm.TU.Chr16.217(UBC2)	UBE2A, UBC2, RAD6A; ubiquitin-conjugating enzyme E2 A [EC:2.3.2.23]
				K10576	evm.TU.Chr16.177(UBC4)	UBE2H, UBC8; ubiquitin-conjugating enzyme E2 H [EC:2.3.2.23]
				K10577	evm.TU.Chr16.435(SCE1)	UBE2I, UBC9; ubiquitin-conjugating enzyme E2 I
				K10583	evm.TU.Chr5.251(UBC22))	UBE2S, E2EPF; ubiquitin-conjugating enzyme E2 S [EC:2.3.2.23]
				K10598	evm.TU.Chr10.687(CYP65)	PPIL2, CYC4, CHP60; peptidyl-prolyl cis-trans isomerase-like 2 [EC:5.2.1.8]
				K10601	evm.TU.Chr19.341(HRD1A)	SYVN1, HRD1; E3 ubiquitin-protein ligase synoviolin [EC:2.3.2.27]
				K10606	evm.TU.Chr10.729(Fancl)	FANCL, PHF9; E3 ubiquitin-protein ligase FANCL [EC:2.3.2.27]
	3	Ribosome	ko03010	K02866	evm.TU.Chr5.337(RPL10))	RP-L10e, RPL10; large subunit ribosomal protein L10e
				K02870	evm.TU.Chr17.737(RPL12)	RP-L12e, RPL12; large subunit ribosomal protein L12e
				K02871	evm.TU.Chr16.73(rplM)	RP-L13, MRPL13, rplM; large subunit ribosomal protein L13
				K02877	evm.TU.Chr12.880_evm.TU.Chr12.881(RPL15)	RP-L15e, RPL15; large subunit ribosomal protein L15e
				K02897	evm.TU.Chr16.400(rplY)	RP-L25, rplY; large subunit ribosomal protein L25
				K02898	evm.TU.Chr10.685(RPL26A)	RP-L26e, RPL26; large subunit ribosomal protein L26e
				K02899	evm.TU.Chr16.51(RPL27)	RP-L27, MRPL27, rpmA; large subunit ribosomal protein L27
				K02911	evm.TU.Chr19.368(--)	RP-L32, MRPL32, rpmF; large subunit ribosomal protein L32
				K02915	evm.TU.Chr16.230(RPL34)	RP-L34e, RPL34; large subunit ribosomal protein L34e
				K02915	evm.TU.Chr19.407(RPL34)	RP-L34e, RPL34; large subunit ribosomal protein L34e
				K02915	evm.TU.Chr19.408(RPL34)	RP-L34e, RPL34; large subunit ribosomal protein L34e
				K02918	evm.TU.Chr17.832(RPL35)	RP-L35e, RPL35; large subunit ribosomal protein L35e
				K02924	evm.TU.Chr16.303(RPL39C)	RP-L39e, RPL39; large subunit ribosomal protein L39e
				K02926	evm.TU.Chr17.924(RPL4)	RP-L4, MRPL4, rplD; large subunit ribosomal protein L4
				K02935	evm.TU.Chr10.693(rplL)	RP-L7, MRPL12, rplL; large subunit ribosomal protein L7/L12
				K02935	evm.TU.Chr16.156(RPL12)	RP-L7, MRPL12, rplL; large subunit ribosomal protein L7/L12
				K02936	evm.TU.Chr16.34(RPL7A-1)	RP-L7Ae, RPL7A; large subunit ribosomal protein L7Ae
				K02939	evm.TU.Chr17.680(RPL9)	RP-L9, MRPL9, rplI; large subunit ribosomal protein L9
				K02950	evm.TU.Chr11.1018(rps12-B)	RP-S12, MRPS12, rpsL; small subunit ribosomal protein S12
				K02957	evm.TU.Chr17.825(RPS15A)	RP-S15Ae, RPS15A; small subunit ribosomal protein S15Ae

				K02957	evm.TU.Chr17.826(RPS15A)	RP-S15Ae, RPS15A; small subunit ribosomal protein S15Ae
				K02957	evm.TU.Chr17.829(RPS15AA)	RP-S15Ae, RPS15A; small subunit ribosomal protein S15Ae
				K02957	evm.TU.Chr17.841(RPS15AA)	RP-S15Ae, RPS15A; small subunit ribosomal protein S15Ae
				K02957	evm.TU.Chr17.844(RPS15A)	RP-S15Ae, RPS15A; small subunit ribosomal protein S15Ae
				K02966	evm.TU.Chr16.280(RPS19A)	RP-S19e, RPS19; small subunit ribosomal protein S19e
				K02976	evm.TU.Chr17.1099(RPS26C)	RP-S26e, RPS26; small subunit ribosomal protein S26e
				K02981	evm.TU.Chr5.274(RPS2D)	RP-S2e, RPS2; small subunit ribosomal protein S2e
				K02992	evm.TU.Chr17.986(RPS7)	RP-S7, MRPS7, rpsG; small subunit ribosomal protein S7
				K02997	evm.TU.Chr16.226(RPS9C)	RP-S9e, RPS9; small subunit ribosomal protein S9e
				K02997	evm.TU.Chr19.423(RPS9C)	RP-S9e, RPS9; small subunit ribosomal protein S9e
A-LBA	1	Zeatin biosynthesis	ko00908	K00279	evm.TU.Chr5.146(CKX1))	CKX; cytokinin dehydrogenase [EC:1.5.99.12]
				K13495	evm.TU.Chr5.133_evm.TU.Chr5.132(ZOX1)	CISZOG; cis-zeatin O-glucosyltransferase [EC:2.4.1.215]
				K13495	evm.TU.Chr5.134(ZOX1)	CISZOG; cis-zeatin O-glucosyltransferase [EC:2.4.1.215]
				K13495	evm.TU.Chr5.135(ZOX1)	CISZOG; cis-zeatin O-glucosyltransferase [EC:2.4.1.215]
				K13495	evm.TU.Chr5.144(ZOX1)	CISZOG; cis-zeatin O-glucosyltransferase [EC:2.4.1.215]
				K13495	evm.TU.Chr5.145(ZOG1)	CISZOG; cis-zeatin O-glucosyltransferase [EC:2.4.1.215]
	2	Cutin, suberine and wax biosynthesis	ko00073	K13356	evm.TU.Chr5.36(FAR2)	FAR; alcohol-forming fatty acyl-CoA reductase [EC:1.2.1.84]
				K13356	evm.TU.Chr5.37(FAR2))	FAR; alcohol-forming fatty acyl-CoA reductase [EC:1.2.1.84]
				K15400	evm.TU.Chr5.33(HHT1)	HHT1; omega-hydroxypalmitate O-feruloyl transferase [EC:2.3.1.188]
				K15400	evm.TU.Chr5.34(PHT1)	HHT1; omega-hydroxypalmitate O-feruloyl transferase [EC:2.3.1.188]
				K15400	evm.TU.Chr5.35(HHT1)	HHT1; omega-hydroxypalmitate O-feruloyl transferase [EC:2.3.1.188]
	3	Selenocompound metabolism	ko00450	K00384	evm.TU.Chr5.128(NTRC)	trxB, TRR; thioredoxin reductase (NADPH) [EC:1.8.1.9]
				K01739	evm.TU.Chr16.250(CGS1)	metB; cystathionine gamma-synthase [EC:2.5.1.48]
				K01760	evm.TU.Chr5.180(At3g57050))	metC; cysteine-S-conjugate beta-lyase [EC:4.4.1.13]
A-DLB	1	Tyrosine metabolism	ko00350	K00276	evm.TU.Chr18.995(maoI)	AOC3, AOC2, tynA; primary-amine oxidase [EC:1.4.3.21]
				K00422	evm.TU.Chr4.217(co-2)	E1.10.3.1; polyphenol oxidase [EC:1.10.3.1]
				K00422	evm.TU.Chr4.219(co-2)	E1.10.3.1; polyphenol oxidase [EC:1.10.3.1]
				K00422	evm.TU.Chr4.221(co-2)	E1.10.3.1; polyphenol oxidase [EC:1.10.3.1]
				K00422	evm.TU.Chr4.222(co-2)	E1.10.3.1; polyphenol oxidase [EC:1.10.3.1]
				K00422	evm.TU.Chr4.223(co-2)	E1.10.3.1; polyphenol oxidase [EC:1.10.3.1]
				K01557	evm.TU.Chr6.108(FAHD1)	FAHD1; acylpyruvate hydrolase [EC:3.7.1.5]
				K18857	evm.TU.Chr6.1448(ADH1)	ADH1; alcohol dehydrogenase class-P [EC:1.1.1.1]

				K18857	evm.TU.Chr6.1450_evm.TU.Chr6.1451(ADH1))	ADH1; alcohol dehydrogenase class-P [EC:1.1.1.1]
	2	Thimine metabolism	ko00730	K01662	evm.TU.Chr11.272(TKT2)	dxs; 1-deoxy-D-xylulose-5-phosphate synthase [EC:2.2.1.7]
				K01662	evm.TU.Chr6.165(TKT2)	dxs; 1-deoxy-D-xylulose-5-phosphate synthase [EC:2.2.1.7]
				K03147	evm.TU.Chr11.287(THIC)	thiC; phosphomethylpyrimidine synthase [EC:4.1.99.17]
				K03147	evm.TU.Chr6.177(THIC)	thiC; phosphomethylpyrimidine synthase [EC:4.1.99.17]
				K20896	evm.TU.Chr6.73(TENA_E)	TENA_E; formylaminopyrimidine deformylase / aminopyrimidine aminohydrolase [EC:3.5.1.- 3.5.99.-]
				K22911	evm.TU.Chr20.970(TH2)	TH2; thiamine phosphate phosphatase / amino-HMP aminohydrolase [EC:3.1.3.100 3.5.99.-]
	3	Isoquinoline alkaloid biosynthesis	ko00950	K00276	evm.TU.Chr18.995(maoI)	AOC3, AOC2, tynA; primary-amine oxidase [EC:1.4.3.21]
				K00422	evm.TU.Chr4.217(co-2)	E1.10.3.1; polyphenol oxidase [EC:1.10.3.1]
				K00422	evm.TU.Chr4.219(co-2)	E1.10.3.1; polyphenol oxidase [EC:1.10.3.1]
				K00422	evm.TU.Chr4.221(co-2)	E1.10.3.1; polyphenol oxidase [EC:1.10.3.1]
				K00422	evm.TU.Chr4.222(co-2)	E1.10.3.1; polyphenol oxidase [EC:1.10.3.1]
				K00422	evm.TU.Chr4.223(co-2))	E1.10.3.1; polyphenol oxidase [EC:1.10.3.1]
